# Iterative free-energy optimization for recurrent neural networks (INFERNO)

**DOI:** 10.1371/journal.pone.0173684

**Published:** 2017-03-10

**Authors:** Alexandre Pitti, Philippe Gaussier, Mathias Quoy

**Affiliations:** ETIS Laboratory, CNRS UMR 8051, University of Cergy-Pontoise, ENSEA, Paris-Seine, Cergy-Pontoise, France; University of Manchester, UNITED KINGDOM

## Abstract

The intra-parietal lobe coupled with the Basal Ganglia forms a working memory that demonstrates strong planning capabilities for generating robust yet flexible neuronal sequences. Neurocomputational models however, often fails to control long range neural synchrony in recurrent spiking networks due to spontaneous activity. As a novel framework based on the free-energy principle, we propose to see the problem of spikes’ synchrony as an optimization problem of the neurons sub-threshold activity for the generation of long neuronal chains. Using a stochastic gradient descent, a reinforcement signal (presumably dopaminergic) evaluates the quality of one input vector to move the recurrent neural network to a desired activity; depending on the error made, this input vector is strengthened to hill-climb the gradient or elicited to search for another solution. This vector can be learned then by one associative memory as a model of the basal-ganglia to control the recurrent neural network. Experiments on habit learning and on sequence retrieving demonstrate the capabilities of the dual system to generate very long and precise spatio-temporal sequences, above two hundred iterations. Its features are applied then to the sequential planning of arm movements. In line with neurobiological theories, we discuss its relevance for modeling the cortico-basal working memory to initiate flexible goal-directed neuronal chains of causation and its relation to novel architectures such as Deep Networks, Neural Turing Machines and the Free-Energy Principle.

## Introduction

Hierarchical plans and tree structures are a hallmark for human language and cognition [1]. But how the brain does to construct and retrieve them dynamically? In the motor domain, Wolpert and colleagues propose that the brain learns the causal structure in sensorimotor circuits (e.g., the hidden parameters of a sensorimotor task) to perform action sequences assembled online based on contextual signals from the environment e.g. for coordinate transform or embodied simulation [2, 3]. For this example, it is argued that the causal structure is encoded directly within the neural representations of cognitive chunks or motor primitives that a working memory can access further to explore and construct off-the-shelf satisfying neuronal chains with respect to the context. This adaptivity in the adult brain and human behavior is hypothesized to be constructed slowly during infant development as Piaget and the tenants of the embodied approach of cognition proposed it [4]. This rises difficult questions on how to learn low-level sensorimotor neuronal rules with causal reasoning capabilities? How to explore the different alternatives in the perceptuo-motor space given a specific context? How to initiate flexible yet goal-directed chains of causation (active causation) [5]?

One candidate mechanism for flexible neural coordination is synchrony. At the neural level, experimental and modelling studies have shown that spiking recurrent neural networks (RNN) can encode temporal relationships by strengthening the synaptic connections between neurons. However, the control of the neurons’ spikes at the millisecond order to propagate information is non-trivial: the spontaneous activity within the network rapidly perturbs the neural dynamics and it is rather difficult then to maintain any stability for controlling long-range synchrony. As a novel idea, we envision the coordination of the spikes’ trains as an optimization problem and instead of controlling directly the firing time of the neurons (i.e., the probability of the neuron to fire or not at a specific timing), we propose to control rather the neurons’ sub-threshold activity (i.e., to find which input value can generate a spike at a specified time). Making an analogy with the butterfly effect in chaos theory, we propose that the tiny control of the neurons’ sub-threshold activity can permit to drive at the mesoscopic scale the spikes’ synchrony; [6–8].

**Fig 1:**
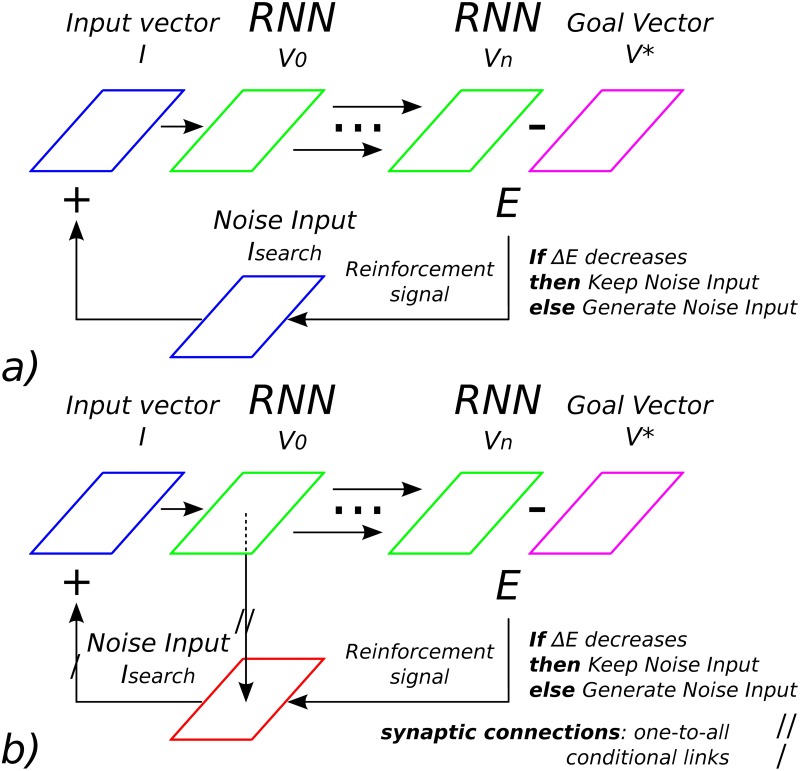
Optimization technique used to control a recurrent spiking neural network. a) Model-free reinforcement signal controls the input vector *I*_*search*_ of RNN by comparing its output vector *V*_*n*_ at time *t* = *n* with respect to a goal vector V*: as *E* is diminishing, the descent gradient stochastically converges to the optimal input vector *I*_*search*_ = I* that generates V*. b) Model-based reinforcement signal, *I*_*search*_ = I* is learned by an associative map and reinjected for any specific *V*_0_.

For this, we propose to use an optimization technique (a reinforcement signal) to drive the neurons’ sub-threshold activity toward a targeting goal; by looping this process several time, we expect the emergence of long-range neural sequences from largely unstructured spiking recurrent neural networks; see Fig 1a). This idea is in line with recent proposals in machine learning, [9] and [10], that use also semi-structured recurrent network models for planning. In comparison to them, we extend their results by adding a second structure along with the recurrent network, an associative map (AM), that will recursively and timely control it; see Fig 1b). We will show that our coupled system can generate long temporal sequences of spikes in a dynamic and robust way recursively. But more importantly, we will explain how this new architecture is now belonging to a different class of algorithms that implements predictive coding [11]. We introduce our model as a neural mechanism based on *Iterative Free-Energy Optimization for Recurrent Neural Networks*, which is the anagram of INFERNO. Moreover, this architecture is supported by several proposals and observations that consider the functional organization between the cortex with the sub-cortical regions (the basal ganglia); c.f. [12–18].

We relate our idea with the principle of Free-Energy minimization (FEM) proposed by Friston [19]. The Free Energy (FE) theory has been proposed to describe a wide range of phenomena such as goal directed behaviour, learning (including habit learning), decision making, e.t.c… Importantly, this theory explains this wide range of phenomena considering the agent embedded in a highly dynamical environment, under the only constraint that the agent has to minimize its free energy. This is a highly encompassing theory which is theoretically well-grounded and based on very general principles. For example, in this context, habit learning is explained as an emergent property of Bayesian optimal behaviour (under Free Energy minimization requirements), by an increased precision of posterior beliefs about future outcomes. That is, this theory provides a conceptualization and a mechanistic account of habit learning.

Free energy scores the evidence that a particular policy is being pursued [20, 21]. FEM means to predict for each policy one expected state and to *optimize* the one that minimizes the most future errors. Implementing FEM has therefore an impact on the architecture design for any predictive systems as it imposes to have at least two systems coupled to each other, one encoding sensory signals and the other predicting its activity. This later system can be considered optimal in Bayesian terms when it can find the hidden causes of the former system and reconstruct its data [22]. Stated like this, FEM relates to other models of cognition such as predictive coding [11, 23, 24], Bayesian population coding [19, 25, 26] or active inference [27–29]. These frameworks promote a hierarchical organization of coupled systems, based on feedback error prediction.

At the brain level, this paradigm is argued to occur at all scales and with different mechanisms, having always an afferent system (e.g., the sensory neurons or the encoder) and an effector system (e.g., the motor neurons but not necessary or the decoder), with the later anticipating the former. In a model of visual processing, Rao and Ballard proposed that the visual cortex is organized hierarchically for encoding natural images based on feedback connections that carry predictions of lower-level neural activities [23]. At the neuron’s level, if this paradigm is also valid, this means that there exists some mechanisms that actively infer for a neuron whether to spike or not with respect to expected incoming signals and corrective error feedback.

**Fig 2:**
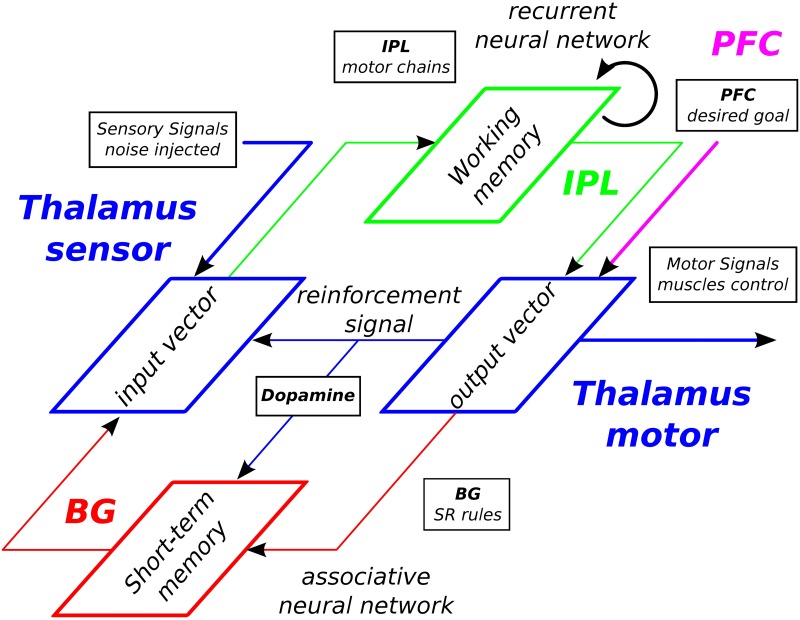
Neural architecture INFERNO for Iterative Free-Energy Optimization of Recurrent Neural Networks. This architecture is a model-free reinforcement learning for exploratory behaviors in a recurrent working memory (WM) of spiking neurons and model-based reinforcement learning in a short-term memory (STM) with reward signal. The former memory model corresponds to the Inferior Parietal Lobe (IPL) where motor chains are assembled dynamically. The later memory model corresponds to the Basal Ganglia (BG) where simple signal-response rules are learned by an associative map (AM) to trigger one spatio-temporal sequence into the working memory. The frontal cortex (PFC) provides the targeting signal to the IPL and BG. The dopaminergic signal supervises both the exploratory search in the WM and the learning in the STM when the goal has been retrieved. RNNs, once unfolded in time, can be seen as a virtually deep feed-forward network in which all the layers share the same weights [33]. The reinforcement signal on the output dynamics can serve to control the input dynamics with noise to search stochastically the inputs that diminish the error to the output dynamics.

INFERNO’s architecture exploits these ideas in spiking recurrent neural networks, having two coupled systems, one inferring the state of the other and trying to ‘correct’ its data based on feedback error. In this formulation, the supervising system (AM) attempts to learn a model of the afferent network (RNN) in accordance to the evidence that a particular policy is being pursued in order to control it for generating long sequences. INFERNO exploits noise (or FE) in spiking neurons for exploring different policies in which the iterative minimizing of prediction errors (or FEM) for one policy augments gradually its expectation. This may correspond to different places in the brain for decision-making and perceptual inference [30]. Here, we propose to link INFERNO to the cortico-basal system for habit formation and sequence retrieving [31], see Fig 2.

In studies on habit formation, several researchers advocate for a dynamic role of the basal ganglia (BG) when interacting with other areas [31, 32]. Although the role of the striatum is commonly focused on the encoding and control of stimulus-response based on dopaminergic reward, Yin and Knowlton in [31] see the BG as a generator of dynamics that selects and amplifies certain dynamics while eliciting others. In their model, information flows from cortex to the basal ganglia to thalamus and back to cortex, but each system is dynamic. INFERNO may relate to these features of the cortico-basal system as it exploits noise as a generator for diversity and error prediction minimization for goal-directed behavior.

In line with this, [16] proposes that the flexible processing of contextual situation done in the neo-cortex (CX) is driven by a sub-cortical controller, the basal ganglia (BG), toward a targeting goal provided by the prefrontal cortex (PFC). We will discuss about the relevance of our model based on neurobiological considerations in the next section.

In order to demonstrate the capabilities of our model for recursivity and boot-strapping capabilities, we will design several experimental setups for habit learning (top-down control) and retrieval phases (bottom-up self-organization) of spiking neurons sequences, and its application to sequential planning of arm movements. We will discuss then the relevance of our model with respect to neurobiological data, its computational power for robotics and AI, neuromorphic hardware implementations, and its affiliation to certain computational principles of the brain proposed by [21, 33–35].

### Neurocomputational considerations and other models

The computational architecture that we have briefly described in Figs 1 and 2 has some neurobiological foundations. At the brain level, one cortical area found important for processing neural chains is the Parietal cortex that includes the Post-Parietal Cortex (PPC) and the Intra-Parietal Lobe (IPL). These structures are hypothesized to form a working memory of action-perception rules [29]. For instance, some experiments show that they serve for embodied simulation like mental rotation or coordinate transformation [36, 37] and for retrieving/generating spatio-temporal sequences [38, 39]. Recently, they have been identified to serve for sequence generation [9] and for self-generated thought [17].

In line with these proposals, we see the spiking RNN in our framework to play the role of the IPL working memory, the associative map to play the role of BG, the PFC to provide the goal task and the reinforcement signal to correspond to a dopaminergic signal; see Fig 2. Following this, the IPL cortical neuronal chains can be assembled dynamically and recursively toward higher-level actions and functions depending on the targeting goal furnished by other brain structures, supposedly the Pre-Frontal Cortex (PFC) and the Basal Ganglia (BG). This architecture appears important for reaching and grasping [40, 41], arithmetic operations [16, 42] as well as language formation. For instance, in the language domain, lexical chains are hypothesized to be constructed dynamically based on a global context and a set of grammatical rules.

Our computational model of IPL-PFC-BG loop captures some of the features of Daw’s model for the representation of complex tasks [16, 43], which embeds in turn some ideas found in classical symbolic AI about tree-search algorithms. As explained by [16], *“at each state, one can choose between one of many different responses, each of which leads to a new state: In this view, behaviour can be modelled as starting at the top-most ‘node’ in the tree, choosing a response ‘branch’, entering a new state, choosing another response, and so on until one has completed the task (hopefully resulting in a reward)”*. Here, branching is done by BG, entering a new state in the cortical working memory until completion of the task given by PFC using a Dopaminergic reinforcement signal.

Moreover, our model is greatly in line with recurrent spiking neural network models using reinforcement signals for sequential planning [9] and [10]. Its capabilities to boot-strap clusters recursively and to retrieve ordinal sequences make it compatible also with reservoir computing methods [44, 45] such as the echo-state networks [46], RNNPB [47] or the dynamical neural fields [48]. Its properties to assemble dynamically neural chunks remind further Genetic Programming optimization of neural networks like NEAT and others [49].

Interestingly, once unfolded in time, its structure can be seen also as a virtually deep feed-forward network in which all the layers share the same weights [33]. Rolfe and LeCun proposed an architecture similar called DrSAE, in which auto-encoders evaluate and minimize the function given by the recurrent map [50]. The INFERNO architecture combines a self-organized structure (IPL) with a supervised one (BG) as the DrSAE architecture. Here, the reinforcement signal on the output dynamics can serve to control the input dynamics to search stochastically the inputs that diminish the error.

This stochastic descent gradient that we employed in RNN can remind the accumulation of evidences process sampled continuously over time of the LIP neurons [51–53]. These neurons show ramping responses inferring latent decision making so that the better the evidence, the larger the amplitude. The decision making can be seen as a random fluctuation Wiener process pressured by time constraints and decision thresholds [54].

## Methods

### Neural units and STDP-like algorithm

We used in the recurrent neural network a variant of the Hebbian equations, the Rank-Order Coding (ROC) algorithm, which grasps well the structure of the Spike Timing-Dependent Plasticity algorithm and of the classical Delta rule in the spatio-temporal domain [55].

STDP has been discovered to modulate the neural activity of temporally related neurons in many brain regions by reinforcing their links. The Rank-Order Coding algorithm has been proposed by Thorpe and colleagues as a discrete and faster model of the derivative integrate-and-fire neuron and of the standard STDP reinforcement learning algorithm [56]. The rationale is that ROC neurons are sensitive to the sequential order of the incoming signals; that is, their *rank code*. The distance similarity to this code, say *rank*(*x*) –, which corresponds to the *argsort* function in Matlab,—is transformed into an amplitude value by the function $f\left(x\right)=\frac{1}{rank(x)}$.

A scalar product between the input’s rank code with the synaptic weights furnishes then a distance measure and the activity level of the neuron. If the rank code of the input signal matches perfectly the one of the synaptic weights, then the neuron fully integrates this activity over time and fires. At contrary, if the rank coding of the signal vector does not match properly the ordinal sequence of the synaptic weights, then integration is weak and the neuron discharges proportionally to it. To this respect, this mechanism captures the intrinsic property of cortical spiking neurons.

The neurons’ output *V* is computed by multiplying the rank order of the sensory signal vector *x*, $f\left(x\right)=\frac{1}{rank(x)}$, by the synaptic weights *w*; *w* ∈ [0, 1]. For an input vector signal *I* of dimension *M* and for a population of *N* neurons (*M* afferent synapses), we have:

$$V_{n\in N}=\sum_{m\in M}\frac{1}{rank(I_{m})}w_{m,n}$$ (1)

The updating rule of the neurons’ weights is similar to the winner-takes-all learning algorithm of Kohonen’s self-organizing maps [57]. For the best neuron *win* and for each element *m* of the current input signal *I* with *m* ∈ *M*, we have:

$$\begin{array}{ccc} w_{m,win}\left(t+1\right) & = & w_{m,win}\left(t\right)+\epsilon \Delta w_{m,win},\\ \Delta w_{m,win} & = & \frac{1}{rank\left(I_{m}\right)}-w_{m,win}. \end{array}$$ (2)

with *ϵ* the learning rate equals to 0.01 in our experiments.

### Free-energy optimization mechanism

**Fig 3:**
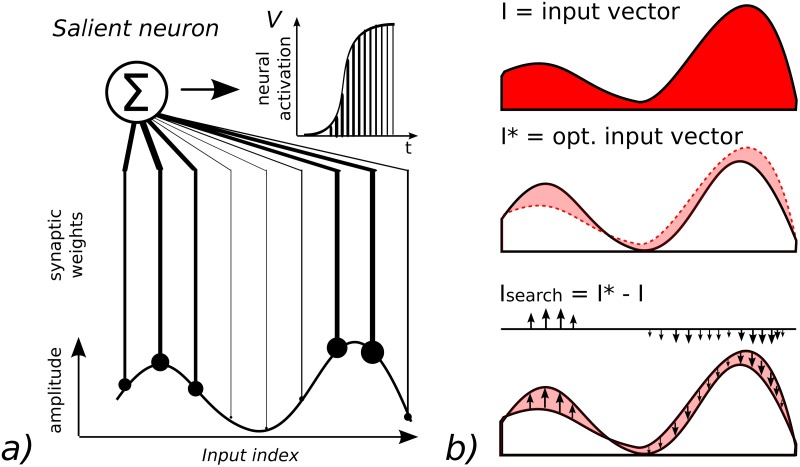
Spike optimization. We can consider the control of the amplitude level *V* of one neuron as an optimization problem. a) For one input vector *I* for which a neuron is the most responsive, we have *I* = I* and *V* = V*. b) Controlling the amplitude level *V* of that neuron requires to find for any input *I*, the input error vector *I*_*search*_ that satisfies *I*_*search*_ = I* − *I*. The exploration of *I*_*search*_ can be done by stochastic gradient descent and meta-heuristics methods. This optimization technique can be applied and extended at a neural population-level.

Viewed as an optimization problem, the control of the RNN dynamics consists in retrieving the most salient inputs that will trigger the neural units to specific amplitude values. This is an inverse problem and can be solved with a gradient descent. In order to explain better the mechanism behind, we can reduce the control of the RNN dynamics to its simplest case with the controlling of one neuron solely, see Figs 1a) and 3a) and 3b).

If we consider I* to be the optimal input signal from which one neuron will fire the most at *V* = V* using Eq 1, one heuristic will consist on searching the term *I*_*search*_ to be added to the current input dynamics *I* so that we can have *I* + *I*_*search*_ = I* and the neuron will reach *V* = V*, see Fig 3b) with *I*_*search*_ shaded in light red. As a meta-heuristic method, retrieving *I*_*search*_ can be done with a stochastic gradient descent (greedy search) by injecting some noise to *I* while using *V* as a metric distance: any intrinsic noise that diminishes the error *E* to the desired goal V* is reinforced and kept (exploitation), or otherwise forgotten to select another random vector *Ie* (exploration). This optimization technique can be extended to a population of neurons and applied to distant rewards, in these cases the terms *I*, *I*_*search*_, *E* and *V* are vectors, see Table 1.

**Table 1:** Free-energy optimization based on stochastic gradient descent to minimize prediction error.

Code	Stochastic optimization as
Lines	Accumulation Evidences Process
*#01*	At time *t* = 0, initialize *V*, V*, *I*
*#02*	choose randomly *I*_*search*_
*#03*	compute *V*_*search*_(*t*) from *V*(*t*), *I* + *I*_*search*_
*#04*	**While** *t* ≤ *horizon_time*, repeat:
*#05*	compute *V*_*search*_(*t* + 1) from *V*_*search*_(*t*)
*#06*	**If** V* − *V* ≥ V* − *V*_*search*_(*t* + 1):
*#07*	*I* = *I* + *I*_*search*_
*#08*	*V* = *V*_*search*_
*#09*	**break**
*#10*	*t* = *t* + 1
*#11*	**Goto** #02

Prediction error *E* on the output vector *V* is used as a reinforcement signal to control the level of noise *I*_*search*_ to inject in the input dynamics *I* in order to explore local or global minima toward V*.

The number of iterations necessary for the WM to converge is not taken into account, therefore the recurrent map will explore several solutions in an unlimited amount of time till convergence. One common solution is to use a threshold value to stop the search. This problem is known in neuroscience as the credit assignment problem [58]: to which particular past event shall we assign credit for the current reward received?

In its present form, the reinforcement signal algorithm corresponds in AI to a classical meta-heuristic method with random walks, which does no prevent from local minima. It may correspond in neurocomputational theory to dopaminergic modulation and to model-free reinforcement learning [59]. However, it does not take into account more sophisticated types of signals, which could be given further by other types of neuromodulators [60].

### Recurrent network model

The neural architecture consists of one recurrent neural network arranged as in Fig 1a). The neurons in the recurrent map (*N* = 25 neurons) encode a temporal sequence directly from their feeded back activity. The temporal horizon *H* for each synaptic link is defined to be of *H* = 20 iterations max (1 *ms* corresponds to 1 cycle), which is therefore the maximum possible time length to be encoded by any synaptic link. Its value is chosen with respect to the average synaptic time found in the neurons of the cortical maps, about 50 *ms* [61]. The network is implemented as a buffer of dimension [*H* × *N*] = [20 × 25] so that each neuron *n* integrates with the synaptic weights *w*_*m*,*n*_ and the function *f*(*BUFFER*[*m*]) with *m* ∈ *M* and *M* = [*HN*] to generate the output value *V*_*n*_. To force the network to be recursive, we update at each iteration the buffer by shifting at each iteration the rows to have *h*(*x* + 1) ← *h*(*x*) and by adding to the first row of the buffer at *h* = 0, the latest update of the neural activity *V*_*n*_, see Table 2. Now, in order to inject external inputs *I* to the recurrent network, the neural population *V*_*n*_ receives an input vector of same dimension *I*_*n*_ added to the first row of the buffer and *only* at *h* = 0 and weighted by 0.5; *V*_*n*_ = ∑ *f*(*BUFFER*)*w*_*m*,*n*_ + 0.5 * *I*_*n*_. The function *f* is the inverse function as explained in section 2.1.

**Table 2:** Description of the buffer algorithm used to simulate integration over a temporal horizon.

Code	Recurrent Map
Lines	Buffer to compute temporal horizon
*#01*	**Compute** *V*_*n*_, *V*_*n*_ = ∑_*m*_ *f*(*BUFFER*)*w*_*m*,*n*_ + 0.5 * *I*_*n*_
*#02*	**Shift** the buffer with *h* ∈ [0, *H* − 1], *BUFFER*[*h* + 1, *n*] ≔ *BUFFER*[*h*, *n*]
*#03*	**Add** to the first row *h* = 0, *BUFFER*[0, *n*] ≔ *V*

The buffer is used to model the recurrent activity of the neural network over time. After each iteration, the buffer that retranscribes the neural activity over time is shifted and presented again to the neural population.

### Associative network model

The previous section explains how our optimization technique serves to retrieve the optimal $I_{search}^{*}$ to be added to the current input vector *I* using the reinforcement signal *E*, the error signal, in order to reach the desired amplitude value V*. The optimal signal $I_{search}^{*}$ found can be learned by an associative layer with perceptrons with all-to-one and one-to-all connections that link the input value *I* to the associative neurons *V*_*a*_ and these to the output value $I_{search}^{*}$, see Fig 1b). The neurons’ equation is similar to the equation of Kohonen neurons with *V*_*a* ∈ *A*_ = ∑_*m* ∈ *M*_ *g*(*I*_*m*_, *w*_*m*,*a*_) (all-to-one connectivity) and $I_{search}^{*}=\sum_{m\in M}g\left(V_{a},w_{m,a}\right)$ (one-to-all connectivity), where

$$\begin{array}{ccc} g\left(x,y\right) & = & \frac{1}{1+RMS\left(x,y\right)},\\ RMS\left(x,y\right) & = & \sqrt{\sum {\left(x-y\right)}^{2}}. \end{array}$$ (3)

The weights of the associative neurons are updated with respect to the reinforcement signal Δ*E* in RNN, similar to Eq 3:

$$\begin{array}{ccc} w\left(t+1\right) & = & w\left(t\right)+\epsilon \Delta E\Delta w,\\ \Delta w & = & I\left(t\right)-w\left(t\right),\\ \Delta E & = & E\left(t\right)-E\left(t-1\right). \end{array}$$ (4)

## Results

We resume in the Table 3 below the different experiments that we have done to present our model. The first experiment corresponds to the study of the RNN optimization along with the stochastic descent gradient toward goal-driven control. The second experiment presents its application to a 3 degrees-of-freedom robotic arm control. The third experiment shows the AM-RNN coupled system and its capabilities for habit learning; e.g., for arm postures. The fourth and fifth experiments describe the ability of AM-RNN working memory to generate long-range spatio-temporal learned sequences, in a flexible way (resp. experiment 4) or in forced fashion (resp. experiment 5).

**Table 3:** Table of the different experimental setups.

Section	Exp.	Architectures
3.1	1	RNN, Optimization control
3.2	2	RNN, Arm control
3.4.1	3	AM ↔ RNN, Habit learning
3.4.2	4	AM ↔ RNN, Bottom-up
3.4.3	5	PFC→AM↔RNN, Top-down

Description of the different experiments done on their corresponding section.

### RNN goal-driven control

In this section, we study solely the RNN, decoupled from the associative map, in order to explain its behavior during goal-driven control. First the recurrent map learns some spatio-temporal rules for several iterations until convergence of its dynamics. This is done using the reinforcement mechanism presented in the previous section. When the neurons’ synaptic weights become stable enough after one thousand iterations, the network is ready to be used for testing.

**Fig 4:**
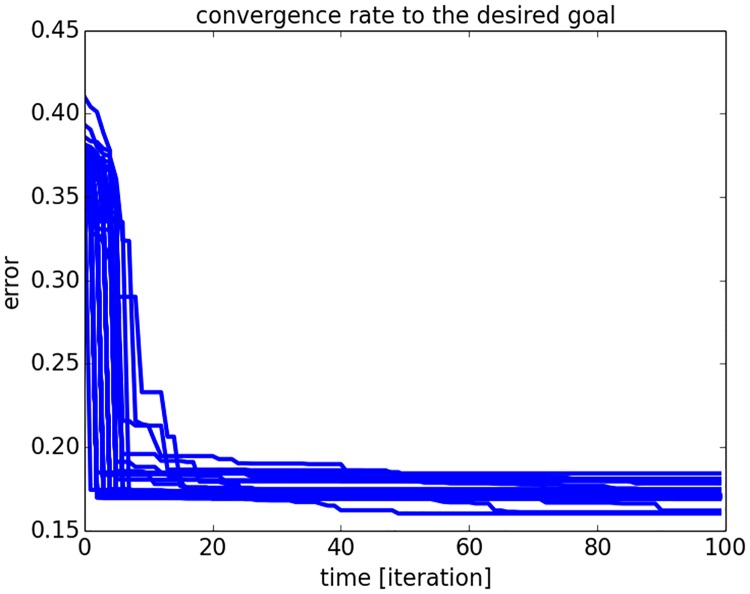
RMS convergence to one targeted goal by the RNN for one hundred trials. The amplitude level of the neurons in the RNN converges to the desired output vector rapidly in dozen iterations; some solutions are more precise than others due to local minima.

**Fig 5:**
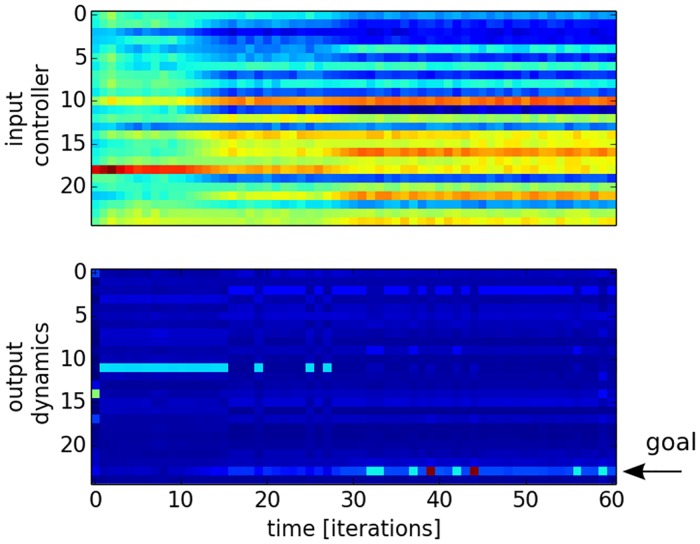
Raster plots of the input vectors injected to the RNN and its respective output vector for the first 60 iterations toward a target solution. Following a hill-climbing random walk, we observe the rapid retrieval by the input controller of the desired RNN’s spatio-temporal pattern (a different example of such desired pattern is presented in Fig 6); the blue color is for a low activity level and the red color is for a high activity level.

For this, we define a desired output V* as goal vector and we let the reinforcement signal drive the search of the input vector *I*_*e*_ from a fixed input vector *I* chosen arbitrarily and only for the first iteration. We plot in Fig 4 the euclidean distance of RNN’s output *V* to the desired output vector V* for one hundred trials starting with different initial conditions. This first graph shows how well all trajectories of the network are converging to a global minima. This convergence is also fast as it requires at most 20 iterations to reach it. We display in Fig 5 the raster plot of the neurons’ dynamics for the input vector in the top chart and for the output vector. After initial conditions, the input and output vectors converge both rapidly to a stable pattern, for which the neuron 24 is the most active neuron (indicated arrow).

**Fig 6:**
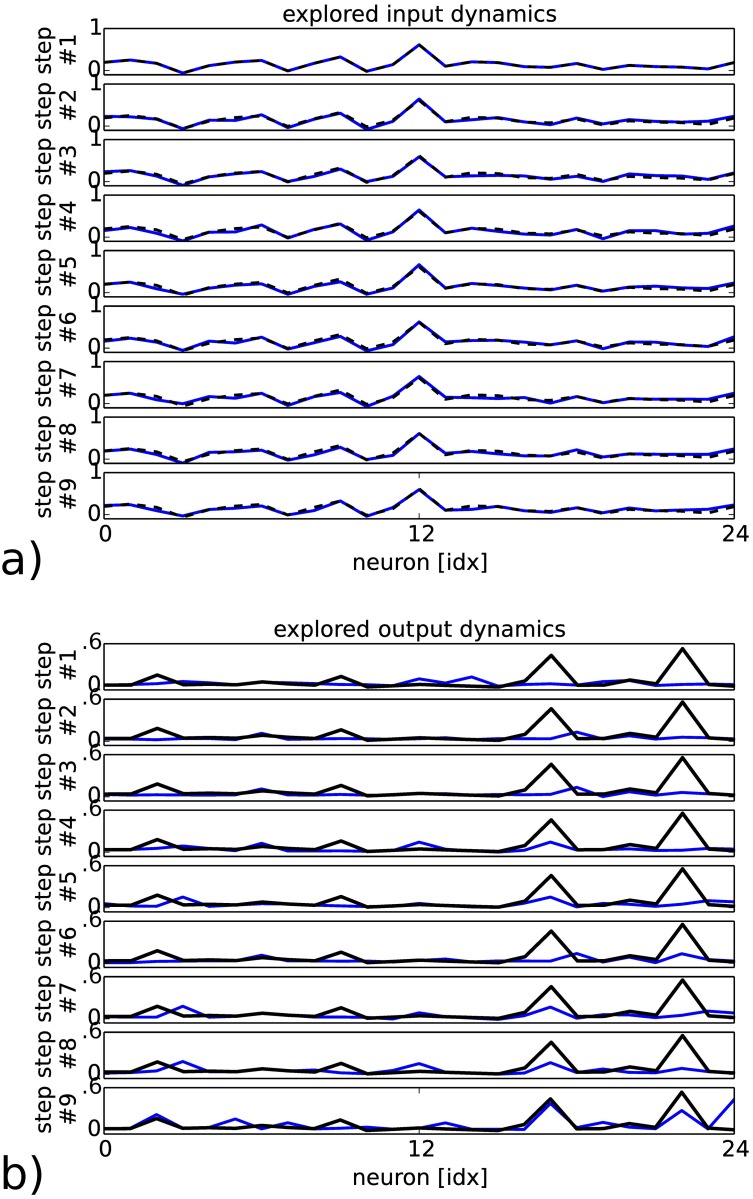
Snapshot of the explored input and output’s RNN dynamics for the first 10th iterations and their convergence to the desired output values (in black); resp. a) and b). The small amplitude variations added in the input dynamics (dashed line) achieve to induce big output changes in RNN with the triggering of the desired spikes (plain black line).

The goal-directed behavior of the working memory is also exemplified in Fig 6a) and 6b) in which the neurons dynamics at several time steps is plotted for the input and output vectors respectively. The super-imposed activity level in black for the input and output vectors corresponds to small variations of the input vector controlled by the reinforcement signal in a) (dashed line) that induce the convergence of the output dynamics to the desired vector in b) (plain line). We observe that *small* amplitude variation in the input dynamics is well sufficient to make *big* amplitude variation in RNN as the output dynamics in blue gradually converges to the desired goal. This shows that the working memory can be controlled as a dynamical system or a chaotic system and its sensitivity to initial conditions can be used to retrieve any spatio-temporal pattern as it would be for an attractor [62].

**Fig 7:**
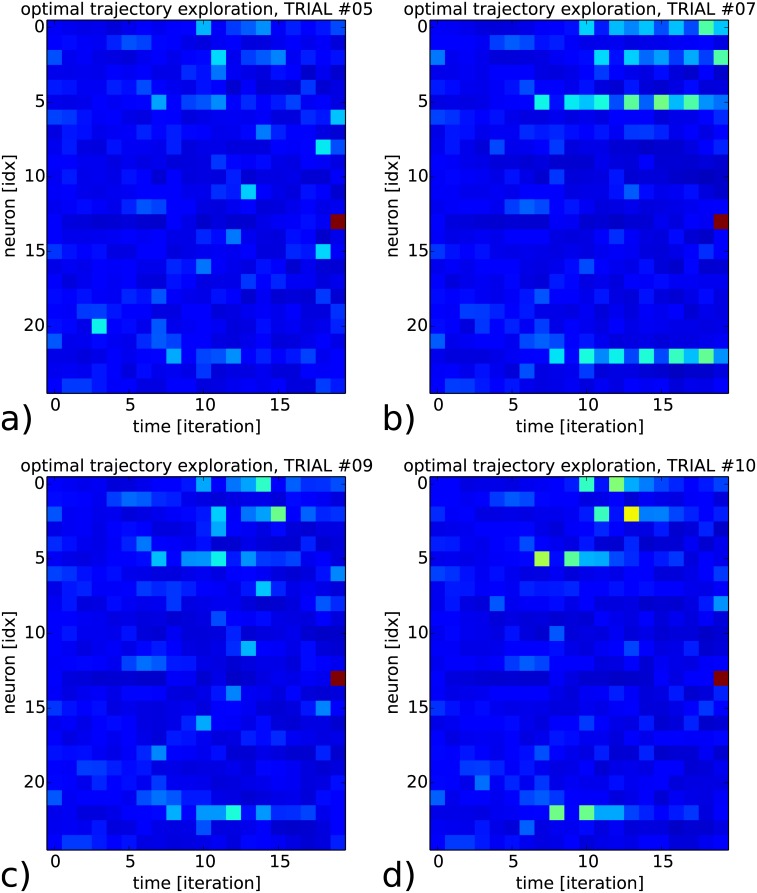
Raster plots of four strategies found by the RNN to trigger the firing of neuron #14 in twenty iterations. The four trajectories show some similar sub-threshold patterns although they exhibit also high variabilities, in the temporal delays as well as in the amplitude level.

In Fig 7 we present four raster plots taken from the recurrent map, which all converge to the same neuron spiking, neuron #14 in red at time *t* = 20 iterations, and for a different goal than in the previous figure. The amplitude level of the recurrent map dynamics for the four maps are different yet they converge all to the triggering of the same neuron. We make the note that the neural activity at the population level is sub-threshold till the activation of the desired neuron at the end. Although the network and the learning process are based on spikes, the inter-dependency among the neurons is enough to produce weak coordinated dynamics, which can have a strong effect.

**Fig 8:**
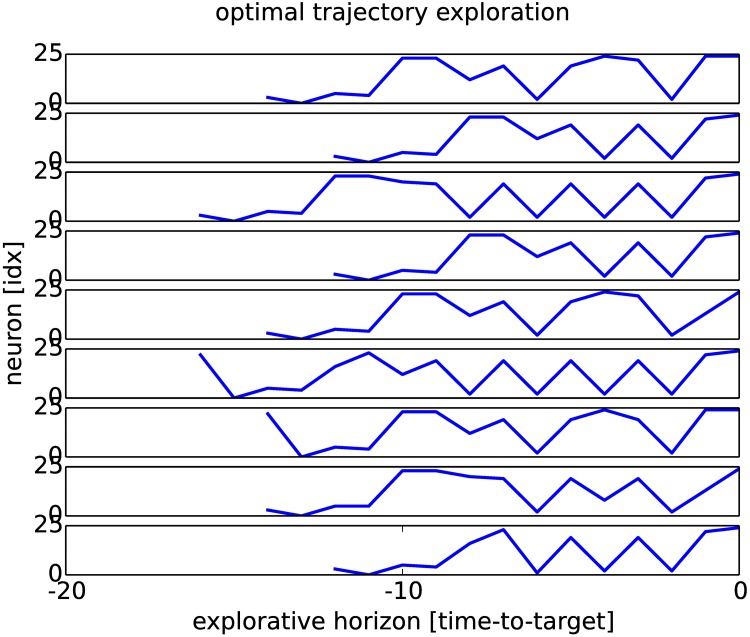
Ten trajectories found till triggering of neuron #25. The trajectories are created by picking up at each iteration the most active neuron. The ten trajectories present a mix of neural chunks common to all trajectories and of novel patterns found solely in them. Each trajectory is retrieved dynamically (novelty) although the solutions appear similar (redundancy) due to the constraint dynamics in RNN.

The causal chaining in the neural network is not straightforward to observe. We propose therefore to plot the spatio-temporal trajectory within the working memory for ten solutions found; see Fig 8. We plot the neural trajectory till reaching the goal vector by selecting at each iteration the most active neuron. In our example, the goal to reach is the neuron #25 ordered from the time-to-trigger = 0 at the most-right hand side of the plot. We emphasize that the most active neuron at each iteration is also the most influential for driving the neural activity for the next steps. We can observe from the graph that all trajectories have different lengths, although in average they converge after ten iterations. At the same time, the spatio-temporal trajectories present some similar patterns within their dynamics placed coherently at the beginning, middle and end of the sequence that we retrieve in different trials. These patterns come from the short-range synaptic rules learned and represent one chunk or one unit that is combined with others to constitute a longer chain, up to sixteen elements in our case. We stress that these chunks are dynamically assembled and not predefinedly learned, although they present one stable shape.

### RNN arm control

**Fig 9:**
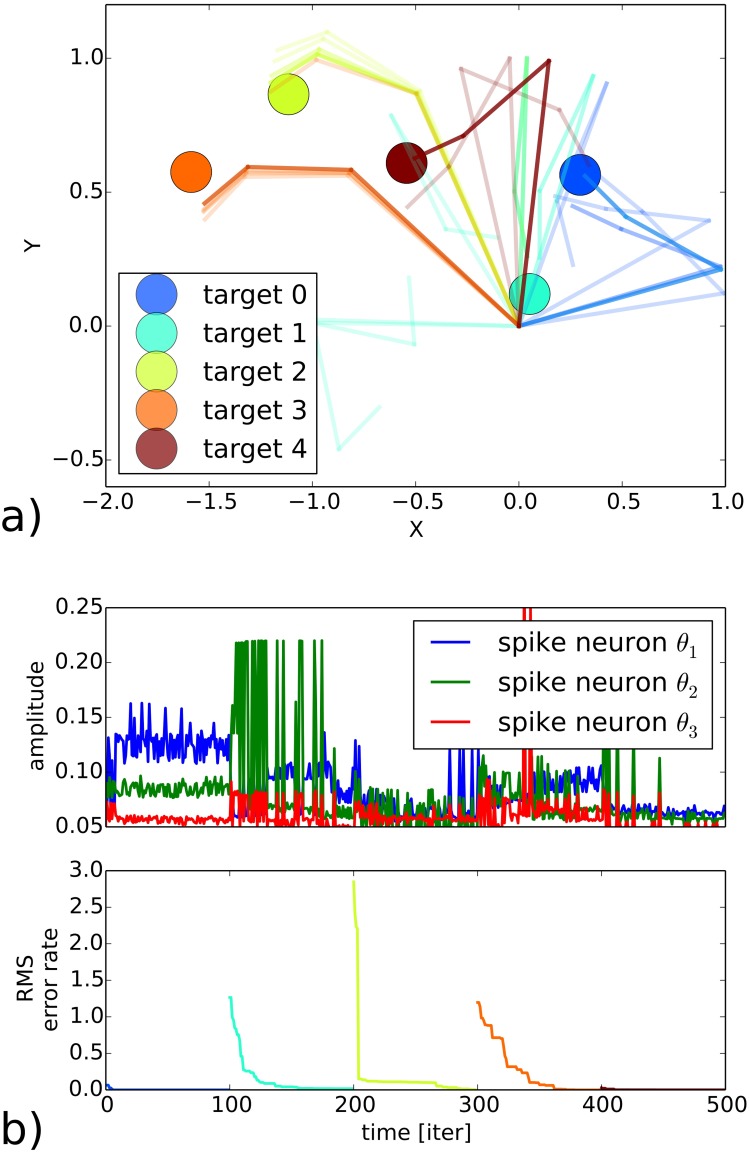
Arm control by the recurrent network with a reinforcement signal. a) The three d.o.f. planar robot is controlled by the spiking recurrent neural network for which the amplitude level of three neurons control the three joint angles. b) The euclidean distance to the goal location furnishes a reward to the motor neurons.

We use the RNN as a working memory for controlling the motion of a three joints robotic arm in a 2D space, see Fig 9a). We exploit the goal-directed behavior of the recurrent network for sequential planning and for the reaching of five positions in space. The three angles of the robotic arm are coupled to the dynamics of three neurons of the recurrent network with same properties than the one presented in the previous section and the reinforcement signal is simply the euclidean distance of the end-effector to the goal. The neural activity between [0; 0.1] for the three neurons (sub-threshold activity) were renormalized between [0; 2*π*] in radians for each joint angle. The result of the arm trajectory is presented in Fig 9a) and the output dynamics of the neural network is shown respectively in Fig 9b). The network easily retrieves the different positions in several iterations and updates its dynamics exploiting the reinforcement signal.

### Spiking recurrent network analysis

**Fig 10:**
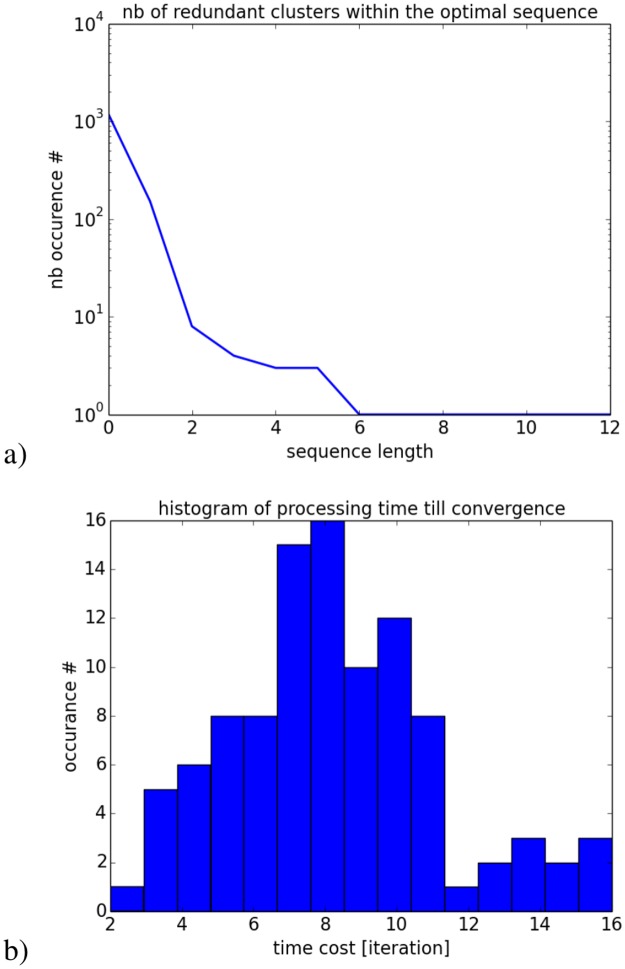
Cluster analysis in RNN. In a), number of clusters found within an optimal sequence with respect to their length. This histogram shows that for any optimal sequences, repetitive clusters are found, which are more often present when they are small than big; this shows some hierarchies within RNN and the property of scale-free dynamics. In b), the average processing time necessary for the RNN to retrieve the goal dynamics. For one hundred trials, it requires in average a dozen of iterations till convergence.

In order to understand better the organization of the spiking recurrent network, we analyze its functional properties at the population level and its dynamics at the neuron level. First, we analyze the redundant clusters found within the optimal sequences and the processing time necessary to discover them, resp. Fig 10a) and 10b). In Fig 10a), we have counted the occurrence of clusters (neural pairs, triplets, etc…) retrieved for a long period of time during spontaneous activity with respect to their size. These clusters are not orthogonal from between each other but are combined into longer-range patterns so that their frequency is inversely proportional to their length; ordinal neural pairs and triplets are proportionally easier to be triggered and retrieved than longer clusters. Meanwhile, the log-curve histogram and cluster coefficients indicate the hierarchical structure of the sequences, which corresponds to scale-free dynamics and small-world properties of the recurrent network [63]. Thus, the reaction time necessary to retrieve one goal depends on the problem complexity (e.g., the locking into a local minimum or not) and requires around ten iterations in order to converge.

In Fig 10b), the reaction time depends mostly on the initial conditions of the recurrent network and of the explorative search. For solutions difficult to retrieve, the map requires an explorative search above a dizain of iterations. This variance can be compared with the density probability found in the real IPL neurons during visual search, which shows similar trends [52].

**Fig 11:**
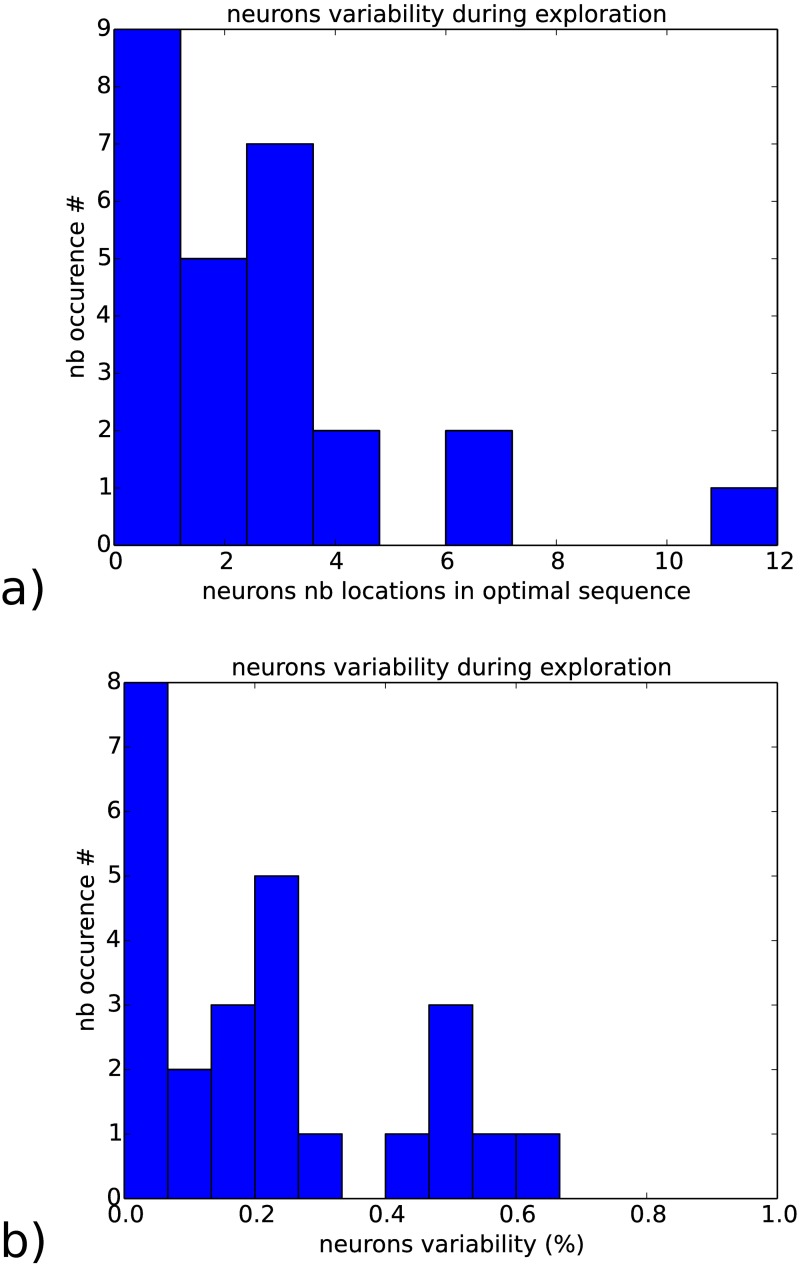
Histogram of the neurons variability measured during exploration and their relative position found within the sequence for hundred trials. These graphs attempts to explain how exploration is done. In a) and b), during the solution-search, the two third of the neurons are rapidly placed within the optimal sequence and one third of the neurons are highly variable and can change positions up to twelve locations within the sequence.

At the neuron level, we measure the probability distribution of the neurons’ variance till convergence to a desired goal since variance is a hallmark of cognitive activity and of decision making [52]. The histogram presents a log-curve distribution with two-third of the neurons with low or weak amplitude variability (variance) and one third of the neurons high amplitude variation, see Fig 11. This results indicates how exploration is done, having one third of the neurons really effective for the neural map to converge to the output dynamics and to generate a spatio-temporal pattern, whereas the rest of the neurons is not.

It indicates also the neurons’ connectivity level within the RNN, or its sparsity. One-third of the neurons interact with each other so that weak amplitude variations in a small set of neurons is enough to interact with another subset and to control its activity. This feature has been emphasized in nonlinear mixed selective neurons [64].

### BG-IPL coordination: Recursivity and bootstrapping

In the previous section, we have investigated the control of a recurrent network by a reinforcement signal mechanism to drive its output dynamics to a desired goal as in Fig 1a). We propose here to complete our architecture and to add an associative map AM that learns the RNN’s input-output association with respect to the reinforcement signal already used for explorative purpose, see Fig 1b). By learning directly the inputs that produce a high-valued reinforcement signal, we can reduce the exploration phase and boot-strap at the same time the working memory dynamics to the goal trajectory. By doing so, we expect the two interacting learning systems to generate longer spatio-temporal sequences of sub-goals. This schema is assumed to be played by the Basal Ganglia, which learns rapidly simple stimulus-response rules, and the IPL-like RNN, working at a slower temporal rate [43]. As an analogy with reinforcement learning, it corresponds to learn the rewarding Q-values associated to an action [60]. In our framework, the Q-values correspond to the activity-level of the AM neurons. This optimization technique in our case can be viewed as model-based reinforcement learning [65].

The bi-directional coupling between the two systems can be done in two ways to generate longer spatio-temporal sequences: in a self-driven fashion when it is the RNN that controls the AM or in a controlled fashion, when the AM controls tightly the RNN’s activity. In our example, this second memory contains twenty neurons so that each neuron can trigger a specific spatio-temporal sequence of the RNN. These two ways are explained thereafter.

#### Habit learning of arm sequence

We propose to re-use the experiment done on arm control in section 3.2 but this time for learning the targeting goals with an associative memory during exploration of the IPL-like recurrent network.

**Fig 12:**
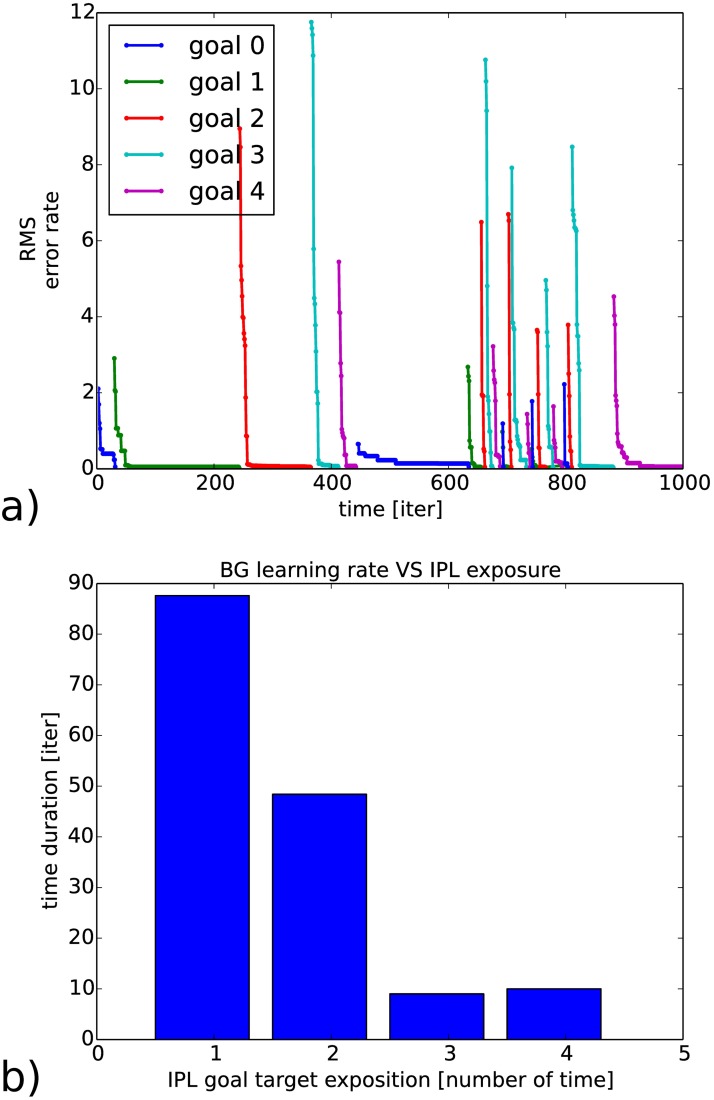
BG training by the IPL neural network and convergence rate with respect to the number of exposure of targeting goals. In a), time duration and error rate for the IPL network to reach the assigned goals iteratively (arm posture). In b), as the associative map learns the recurrent map inputs, the convergence rate decreases on average with the number of exposure to the goals.

We present in Fig 12 the averaged learning rate and convergence time when the BG-like associative network is exposed to several presentation of the same goals; respectively in a) and b). We can observe that the average time interval required by the associative map to make the IPL map to convergence is decreasing for each exposure of the targeting goal: as the BG network is learning, the explorative search done on the IPL dynamics is diminishing over time, see Fig 12a). Sometimes however, the error level appears not related to the number of exposure as for the blue curve around iteration 500 for example because we might be in a local minima, which makes the error correction to be slow. Nonetheless, the recurrent network trains the associative network faster and the response time to retrieve any sequence is quicker, see Fig 12b). Without the BG network, the response level would have been slower and similar to the level found at its slowest performance as during the first exposure.

#### IPL→BG→IPL bottom-up associative recall

**Fig 13:**
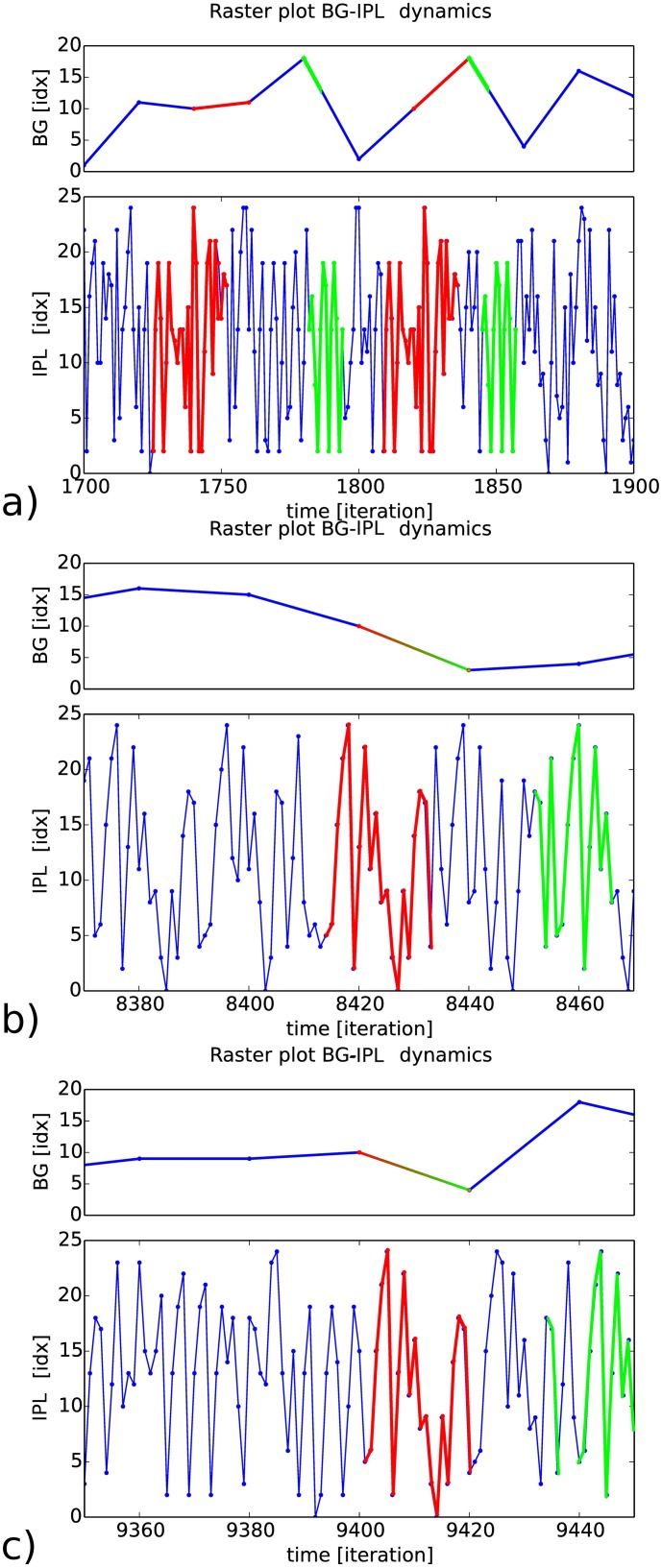
Interactive coupling between the recurrent map and the associative map. Each neuron of the BG-like associative memory learns a stimulus-response pattern that triggers a specific spatio-temporal pattern in the RNN IPL-like working memory. In a), we super-imposed in red the RNN dynamics when the AM neuron #10 triggers. Stable spatio-temporal clusters as long as 28 iterations can be retrieved. The AM BG-like network can bootstrap dynamically the neural population of the IPL-like network. In b) and c), when two consecutive AM/BG pairs are formed –, here the neurons #10 and #3,—the RNN/IPL network can form longer sequences although they possess some variability within it; these longer sequences (40 iterations) are above the temporal horizon of each neuron, which is of 20 iterations.

When we let the two coupled systems work in an autonomous fashion –, which means that we do not force the activity of one specific AM (BG) neuron for example,—the RNN’s output activates the most salient neuron in the AM BG-like network, which recursively controls the RNN’s dynamics in return, see Fig 13a). The result is the autonomous recall in a self-organized fashion of spatio-temporal patterns by the AM BG-like neurons of the exact RNN ordinal sequence –, in our case of thirty steps,—so that when one BG neuron is activated, its corresponding sequence is observed; e.g., the two same sequences reactivated are super-imposed in red.

As similar to the RNN neurons, the BG-like neurons can form also spatio-temporal sequences to create longer patterns. When the same pair is activated as in Fig 13b) and 13c) in red and green traces, the slightly same sequences in RNN are reproduced. The activation of these two chunks can be considered as part of one integrated sequence over an interval span of forty steps.

**Fig 14:**
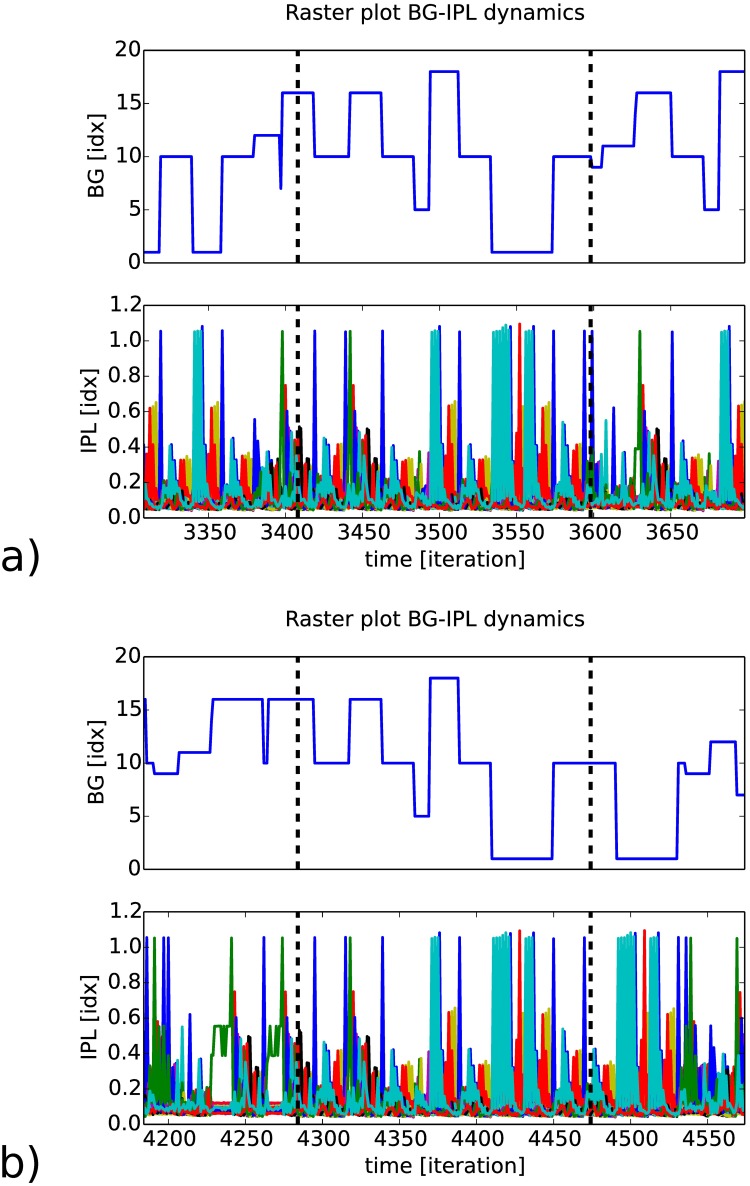
Self-driven interaction between RNN (IPL) and AM (BG). Presentation of the amplitude dynamics of the recurrent map for a sequence length of two hundred iterations between 3420 and 3600 in a), and 4280 and 4460 in b). The amplitude level of the recurrent map is almost similar within the interval of the two dashed lines in black.

In certain situations, when the two maps have a very stable bi-directional coupling, the coupled systems can generate even longer sequences above 190 iterations, see Fig 14. In this figure, the raster plots taken at two different period of time are almost aligned from each other within the black dashed lines. The associative map has generated a sequence over ten neurons.

#### PFC→BG↔IPL top-down control, forced bootstrapping

Self-driven activity shown in the previous section can generate long range episodes, but can we generate even longer ones by forcing the temporal order of AM neurons activation? This experiment differs from the previous one in the sense that we externally force the activation of BG-like neurons to fire in a specific order: i.e., we bypass the spontaneous activity of AM neurons and we control the one selected till convergence of RNN to the desired output dynamics, which means till the AM neuron activity is satisfyingly high above a threshold. This role is supposed to be played by the PFC, which can learn the sequential order of the AM BG-like neurons. This feature will not be investigated in this paper.

**Fig 15:**
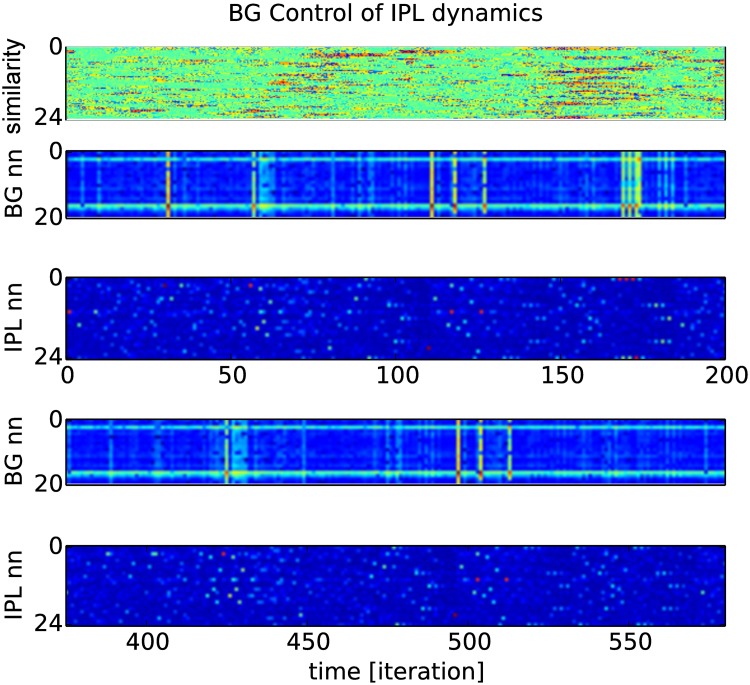
Open-loop IPL control by the BG-like neurons following an ordinal sequence. Every twenty iterations corresponding to the temporal horizon of the IPL buffer, the IPL dynamics are bootstrapped from the AM neurons activity. In our example, the same AM (BG) sequence is injected to the RNN (IPL) dynamics for the two raster plots at different periods of time. The comparison between the two dynamical systems shows an extreme stability to drive the RNN dynamics over long period of time, even without feedback, see the similarity measure at top.

At each retrieval of one RNN episodic memory, which can be more or less rapid, the next BG neuron is selected in the sequence when its activity level reaches a threshold value, therefore the temporal interval can fluctuate for each episode. Fig 15 presents the forced RNN spatio-temporal sequences at two different temporal intervals for the same serial order activation of the BG neurons. In this figure, the spatio-temporal patterns produced are spanning a very long interval range, over several hundred iterations, which is higher in comparison to the self-driven activity presented previously.

Fig 15 presents the activity control of the AM neurons at two different time intervals (bottom and top charts). This result shows how the spiking order can be stabilized over long spatio-temporal patterns (200 iterations) even within a recurrent map for the generating of neural chains proper to the configuration of the RNN. The similarity measure computed above is based on a co-variation measure to detect the relative temporal displacements between patterns of the two intervals. The AM BG-like neural system ‘replays’ or reenacts the neural chains proper to the one learned during action, as described in simulation theory of action representation [66].

We can compare the two behaviors of the AM-RNN / IPL-BG system by measuring the density probability distribution of the number of clusters found with respect to the clusters’ length, when the two maps are bidirectionally coupled and self-driven (section 3.4.2) or when the activity of the BG map is supervised (section 3.4.3).

**Fig 16:**
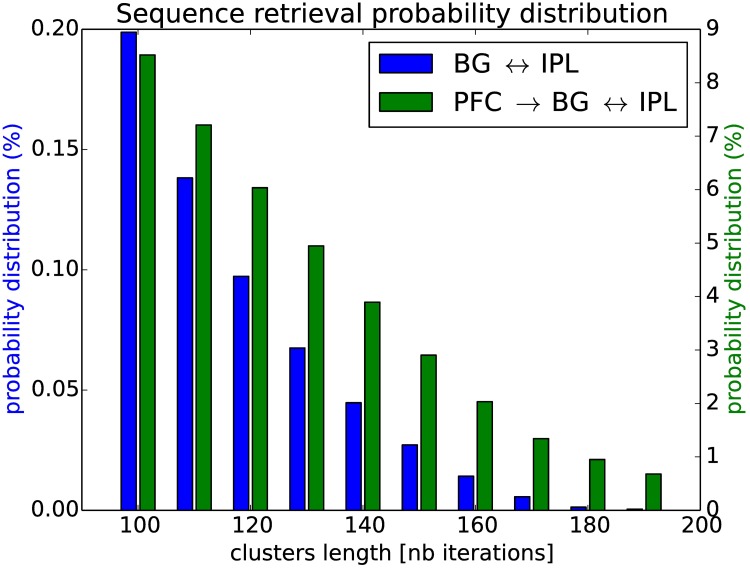
Sequence length retrieval during self-driven and forced conditions. We counted the number of temporal sequences found over time and we computed their probability distribution with respect to their length. In the self-driven condition (in blue, left axis) as done in section 3.4.2, the working memory can repeat spatio-temporal sequences of a maximum length of 200 iterations, which is already above the limits of a conventional spiking RNN. In the controlled condition (in green, right axis) as done in section 3.4.3, the coupled system can retrieve a magnitude longer sequences.

The Fig 16 presents this result with the density probability of the number of clusters found during the self-driven case plotted in blue using the left axis and found during the controlled case plotted in green using the right axis. The two densities present a logarithmic curve of different magnitude order, the self-organized case in blue can generate long range sequences at most of 180 iterations (below 10^−2^%). In comparison, during the controlled case, for which the order magnitude is ten times higher, the probability of occurrence of one sequence of 180 iterations achieved to be reproduced is below 1%. Although robust, the working memory in the self-driven case present more variability and flexibility, which is more advantageous in unexpected situations. Besides, the external control of the associative map (green line) limits strongly the variability of RNN dynamics and induces the reproduction of long-range spatio-temporal sequences as noise is reduced.

## Discussion

We propose a framework based on a coupled recurrent spiking neuronal system that achieves to perform long sequential planning by controlling the amplitude level of the spiking neurons through reinforcement signals. The AM-RNN coupled system exploits error prediction reward to model the cortical and sub-cortical interaction found between IPL and BG networks for neuronal chaining [41] [42]. The control done is weak so that the propagated reinforced signals let the working memory plastic enough to converge to the desired internal states from various trajectories. Used in a robotic simulation, the neural dynamics can drive a three d.o.f. arm to reach online different locations.

The neural control is done below the neurons’ spikes and the sub-threshold amplitude variations injected into the recurrent network can iteratively change its dynamics to make it to converge to attractors or to make it to diverge from repellors. To this respect, our framework embodies some aspects of the free-energy optimization principle proposed by [24] as an optimization technique and some aspects of chaos control of neural dynamics, like *chaos itinerancy* [6, 62, 67], in which small feeded back perturbations can give rise to big amplitude variations and permit to go from one memory to another [68] [69]. It shows also the importance of slow dynamics that persist for a long period of time, which links to critical slowing that is a necessary aspect of free energy minimisation—and links usefully to self-organised criticality [70–74]. At another degree, it conveys also ideas in line with belief propagation and inference in spiking recurrent networks within the Bayesian framework [26] in which the iterated computation embedding the exploration/exploitation stages can be seen as an inference process using reinforcement learning. The free energy optimization process has been proposed to drive flexible neural dynamics in a seemingly coherent manner following the Bayesian paradigm [21, 29].

The functioning of our architecture is partially similar also to recent proposals for sequence generation by [9] and [10], reservoir computing and echo states methods by [44, 45, 75] and to DrSAE model used for classification where auto-encoders iterate a recurrent map using gradient descent [50].

The original distinction of our approach with these techniques resides *(1)* on the control of the neurons’ amplitude to indirectly control the spikes timing, and *(2)* on the use of an extra memory (BG) that learns to associate the correct input vector to inject to the working memory with respect to its output from a reinforcement signal; these two features enable to drive the working memory into a desired state. Its computation can be viewed also as a neural ‘router’ [76] that makes the recurrent network virtually *deep*: i.e., using the output of the recurrent network as its own entry for processing the next stage [33, 50]; e.g., over 200 iterations of virtual layers in Fig 15. For these reasons, the INFERNO compound system has the features of a recurrently deep spiking neural network.

### Computational power

Taking account of the computational power of Rank-Order Coding spiking neurons [55], each neuron can encode 2^*N*^ different representations with *N* their input dimension, in our case *N* = *M* * *O*, with *M* = 25 the size of the neurons’ population and *O* = 20 the temporal horizon of each neuron (i.e., fixed by the buffer length). Besides, each neuron of the associative memory encodes virtually only one trajectory of the recurrent map as a stimulus-response rule; in our case the number of neurons in the associative map is *L* = 20. Therefore, the maximum theoretical length for a spatio-temporal pattern possible to retrieve is equal to *L* * *O*, which is in our case of 400 iterations (or layers). These orders are empirical, however, adding more AM neurons should highly increase the length of RNN sequences produced and the number of possible combinations.

#### Subsumed and complementary systems

As there is evidence that suggests that although single actions can be selected without basal ganglia involvement, chains of actions seem to require the basal ganglia [77]. The BG with the parietal cortex are found both complementary for action planning [41, 45, 78, 79], motor simulation [66] and thought generation [17]. The parietal cortex, involved in implementing complex predictive models as multi-step state-action-state maps (model-based RL), and BG (model-free RL) form a cooperative system driving online behavior [15, 18, 80]. The BG network in our model helps to create long neuronal chains dynamically in the IPL working memory while the IPL working memory trains the BG network.

**Fig 17:**
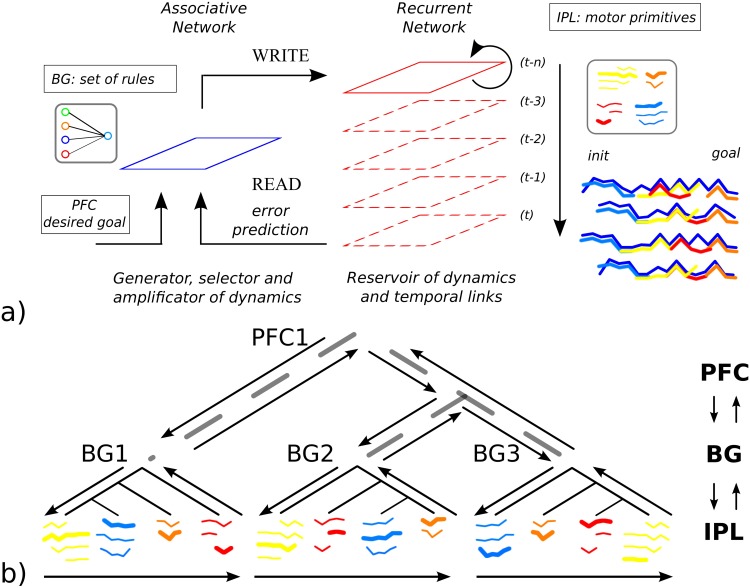
Neurocomputational and AI principles of INFERNO for Working Memory. In a), INFERNO generates, selects and stores a set of rules to assemble dynamically a neuronal sequence from a reservoir of dynamics toward a desired goal and based on free-energy minimization. It has some similarities with a Turing machine that has a table of instructions, Write and Read heads to generate a code from an infinite tape. We super-impose with different colors the clusters of four optimal trajectories found in Fig 8. b) unwrapped in time, INFERNO generates tree-like trajectories as a A* algorithm and as a virtually deep feed-forward neural network.

The numerical limit to subsume new memory maps, one layer at the top of another, is not clear in our model but a third complementary memory, the PFC, can play this role by learning and directing the BG sequences at a higher-level, see Fig 17. This architecture can be replicated hierarchically in INFERNO with many maps inter-connected through continuous feedback control with top-down and bottom-up dynamics [21, 29, 81].

In our model, we have limited the function of PFC to provide one goal at a time so that AM sequences can be formed dynamically in a self-organized fashion along with RNN, see section 3.4.2. Learning this temporal sequence by a top layer can permit to generate an even longer plan execution as done in section 3.4.3 for one sequence only and without any learning. Hence, our model can be extended to a more elaborated PFC model as it is known that PFC contributes to sequential planning over seconds [82] and to the selection of neural ‘programs’ [76].

### Multi-step computation

While the IPL working memory provides, stores, and manipulates representations; the basal ganglia model maps current states to courses of action [83]. BG can serve for selection of complex, sequenced actions at the cortical map level [13]. Thus, it can be interpreted as a repertoire of *if-then* rules or a set of stimulus-response associations to select appropriate cortical chains. In section 3.4.2, we used our cognitive architecture for iterating a long sequential pattern of 200 steps, a serial WM task, which is a feature that can be used for computational purposes (e.g., arithmetic counter). Here, the BG rules can be seen as *’pointers’* of cortical *’programs’*. This kind of cortical architecture has been emphasized to be used possibly for multi-step computation; i.e., for implementing neuronal Turing machines [35, 84–86].

Making an analogy with Turing machines, we can see AM as an instruction table, its operations as the injected inputs into RNN, RNN as the infinite tape and their respective neural activity as symbols and states, see Fig 17a). In INFERNO, we can interpret the execution of one neural rule as follows: **IF** current activity (symbol) in RNN (tape) is j **AND** current activity (state) in AM (instruction table) is i, **THEN** inject the signal k to j (replacing operation).

Furthermore, the reinforcement signal used here as a heuristic function makes a link with more classical AI algorithms using meta-heuristics like the A* tree search, as proposed by Daw [43]. These meta-heuristics are optimization techniques that let the recurrent spiking neural network converge to specific trajectories with some flexibility, see the schema in Fig 17, which are directly taken from the trajectories found in Fig 8. On the one hand, all the trajectories derive from the spatio-temporal primitives learned by the RNN. On the other hand, they are assembled flexibly to reach one goal. Therefore, for each specific goal, the trajectories found in each structure possess roughly the same structure and prototype (global coherence), see Fig 17a) while the structure within each sub-cluster is however different (internal variability), see Fig 17b).

This shows the capabilities of the RNN to produce hierarchical plans and tree structures, which are found important for human language and cognition [1, 87, 88]. Its structural organization follows also a complex network topology as the log-curve distribution of the clusters’ size demonstrate it with scale-free dynamics.

### Gain modulatory control

Our optimization technique is based on the control of the sub-threshold activity of the neurons. We propose that this mechanism can be one candidate for flexible neural coordination, along with phase synchrony and spike timing-dependent plasticity.

For instance, sub-threshold activity optimization is similar to the phenomenon known as gain-modulation [89, 90]. This mechanism describes how the activity level of gain-field neurons can be modulated by the amplitude-level of several neurons sensitive to different variables, which is therefore interesting for neural control [91] and context switch [11]. Gain-modulation is found important for the neural processing in the parieto-motor cortices [92] and may provide a hint on how generative causal chains are formed in a neural population for planning in PFC as proposed by [1].

Gain-modulation has been proposed recently to control the amplitude-level of a neural population (its local field potential). It conveys contextual information in a complex form of propagated neural activity; a mechanism coined as nonlinear mixed selectivity [64]. Furthermore, Botvinick and Watanabe proposed a prefrontal model based on gain-field neurons showing their ability to recall serial order information [90]. Their model assumes that abstract ordinal information is conjoined with item-specific information through a gain-field mechanism.

### Dopaminergic optimization

The neurons in the recurrent network have sparse connections to each other so that the system possesses a high number of spatio-temporal patterns and requires several steps to reach the desired configuration; this behavior corresponds to the characteristics of one working memory. Therefore, in order to retrieve one desired spatio-temporal sequence, a reinforcement signal (presumably dopaminergic neuromodulation) evaluates the exploratory search of the working memory to the desired goal; depending on the reward value, the sensory input dynamics are strengthened to hill-climb the gradient or elicited to search for another solution [93]. This is similar to model-based reinforcement learning for which the internal primitives of the RNN corresponds to the model. Thus, the neural sequences found in Fig 8 are not completely random but depends on the synaptic organization of the RNN so that the later plastically self-organizes to generate the beginning, middle and end of one complete sequence, see Fig 17 on which we super-imposed colours for each stable clusters, as well as cliques and loops found.

### Neuromorphic computation and symbolic AI systems

In comparison to computer memories, the human Working Memory has developed the ability to deal with uncertain data sets and to initiate flexible and robust decision making. Next generation of neuromorphic architectures of spiking neurons based on the functional organization of the brain will be able to mimic the attributes of the Human Working Memory to learn, predict and generate sequences, this will have major impact in the design of next generation computers and autonomous devices such as robots [34, 94].

Different concepts of AI have been applied to the understanding and the modeling of brain functions. Nonetheless, their use in large scale recurrent spiking neural networks is not trivial. INFERNO is an attempt to design a cognitive architecture based on predictive coding and free-energy minimization for categorizing external inputs prone to uncertainties, for generating and stabilizing long-range sequences. It is based on previous neural architectures that we have implemented for modeling working memories. Important concepts that we have borrowed from them are the self-organization of cortical associate maps in an unsupervised fashion based on STDP [95], a novelty-detection mechanism for cumulative learning in a hippocampus model [96, 97], and a taboo greedy search to model the anterior cingulate cortex for error-based exploration [98]. This work and others may help to converge toward some distinct concepts found in classic AI and neural networks to model brain-like cognitive systems [99].
